# PEG-assisted Sol-gel Synthesis of Compact Nickel Oxide Hole-Selective Layer with Modified Interfacial Properties for Organic Solar Cells

**DOI:** 10.3390/polym11010120

**Published:** 2019-01-11

**Authors:** Jung Kyu Kim

**Affiliations:** School of Chemical Engineering, Sungkyunkwan University (SKKU), Seobu-ro 2066, Jangan-gu, Suwon 16419, Korea; legkim@skku.edu; Tel.: +82-31-299-7254

**Keywords:** NiO, charge transport, ITO-free, bulk-heterojunction, organic photovoltaics

## Abstract

As a *p*-type metal oxide, nickel oxide (NiO) has been extensively utilized for providing a favorable hole transport pathway in organic solar cells (OSCs). To obtain higher crystallinity, a post-annealing process at high temperature is required for the NiO layer. Therefore, fluorine-doped tin oxide (FTO) glass has been widely used for the substrate of NiO. However, the rough surface of the FTO substrate deteriorates the interfacial properties of the NiO layer, which hinders efficient charge extraction in OSCs. In this study, a facile polyethylene glycol (PEG)-assisted sol-gel synthesis of the compact NiO layer as the hole-selective layer is demonstrated. The compact NiO layer has a significantly uniform and smooth surface morphology, facilitating better interfacial properties for favorable charge transport. The modified interfacial properties outstandingly promote the charge migration and recombination blocking in OSCs. In addition, a hybrid structure with compact NiO and poly(3,4-ethylenedioxythiophene) polystyrene sulfonate (PEDOT:PSS) is designed to form a cascade charge extraction and passivate possible pinholes on the NiO layer. Consequently, the compact NiO layer enhances all the parameters determining the power conversion efficiency, including the open-circuit potential (*V*_oc_), short-circuit current density (*J*_sc_), and fill factor (*FF*).

## 1. Introduction

The use of the charge transporting interlayer with high charge selectivity is paramount for achieving high-efficiency thin-film organic photovoltaics (OPVs), in which the active layer is composed of bulk-heterojunction (BHJ) with conjugated polymers (donors) and fullerene derivative-based acceptors or non-fullerene acceptors [1,2,3,4,5,6,7]. Since the power conversion efficiency (PCE) strongly depends on the dissociation and recombination of the photo-induced charge carriers, it is critical to develop OPVs with efficient interlayers for balancing the charge extraction behavior from the active layer to each charge collector [8,9,10,11]. Considering the hole migrations in OPVs with conventional structure type, the injected holes at the interface between the BHJ and the hole transport layer (HTL) are transported through the HTL and eventually transferred onto the transparent conductive oxide (TCO) substrate [12]. In recent decades, the combination of poly(3,4-ethylenedioxythiophene) polystyrene sulfonate (PEDOT:PSS) as a hole transporting material and indium-doped tin oxide (ITO) sputtered glass as a TCO substrate has been tremendously exploited for high-efficiency OPVs. However, the acidic property of PEDOT:PSS (~pH 2) dissolves the heavy-metal components in ITO, deteriorating both the long-term stability and initial device performance of OPVs [13,14]. Furthermore, the increasing demand for rare metals such as indium has led to an increase in the price of ITO over time. At present, ITO alone contributes to more than 50% of the cost in the entire process of the OPV module fabrication [15,16]. As an alternative, fluorine-doped tin oxide (FTO) can be used to replace ITO due to its low material price, high thermal stability (up to 500 °C), and better electric conductivity with a given transparency in the visible region of the spectrum (~7 Ω/sq. with ~80% transmittance), of which the resistance value is almost half that of ITO [17,18,19]. However, the high surface roughness is a major obstacle in using FTO. Considering that the size of a single granule of PEDOT:PSS has a diameter of 20–50 nm, the roughness of FTO is too high to form a uniform PEDOT:PSS layer; the root mean square (RMS) roughness of FTO is of the order of a few tens of nanometers [20,21].

As a *p*-type transition metal oxide, nickel oxide (NiO) is a promise hole transporting material [22]. Its low work function (approximately −5.0 eV) and favorable band edges (i.e., −1.5 eV for the conduction band minimum (CBM) and −5.4 eV for the valance band maximum) facilitate the cascade hole transportation with high selectivity, preventing electron-hole recombination [12,23]. Moreover, NiO has a good *p*-type conductance, originating from the quasi-localized holes on Ni^2+^ vacancies in the NiO lattice structure, which generate Ni^3+^ ions [24,25]. Therefore, the nonstoichiometric NiO with nanocrystallinity combining Ni^2+^ and Ni^3+^ is preferred for an efficient HTL. For this, a post-annealing process is followed for the deposition of NiO via solution processes, such as spray or spin-coating methods [26,27]. Thus, FTO has been extensively used for the substrate. However, the challenge that remains in the use of FTO is overcoming its high surface roughness. Its rough surface can deteriorate the morphological and interfacial properties of the HTL and the contact resistance, eventually resulting in the poor hole extraction.

This study demonstrates a facile polyethylene glycol (PEG)-assisted sol-gel synthesis to fabricate a compact NiO layer with nanocrystallinity as the hole-selective HTL. The compact NiO layer has a significantly smooth surface morphology, providing better interfacial properties for favorable charge separation and extraction performances. The modified interfacial properties promote outstanding charge migration and prevent the undesired charge recombination in the OPVs. Moreover, a hybrid structure with compact NiO and PEDOT:PSS was designed to facilitate the cascade charge migration and passivate any possible pinholes on the compact NiO layer. Consequently, the compact NiO layer enhanced all the parameters determining the power conversion efficiency, including the open-circuit potential (*V*_oc_), short-circuit current density (*J*_sc_), and fill factor (*FF*). 

## 2. Materials and Methods

### 2.1. Fabrication of Compact Nickel Oxide Layer

The facile PEG-assisted sol-gel process was carried out to fabricate the compact nickel oxide layer. The nickel oxide precursor solution with 0.2 M concentration was prepared by dissolving nickel chloride hexahydrate (Aldrich) in 2-methoxyethanol (Aldrich). After vigorously stirring at 600 rpm with a magnetic stirrer for 1 h at 60 °C, the pH of the precursor solution was adjusted to 12 by using KOH. 0.2 M polyethylene glycol (PEG, *M*_n_ 300, Aldrich); 0.1 M *α*-terpineol (Aldrich) were added in sequence with simultaneous vigorous stirring. Then, the solution was aged for 24 h with mild stirring (~200 rpm) at 60 °C. For the sake of excluding undesired residuals or microparticles, the solution was filtered using a 200-nm porous cellulose syringe filter. The nickel oxide layer was spin-coated on the pre-cleaned fluorine-doped tin oxide (FTO) glass substrate and annealed at 500 °C. Before the spin-coating process, the surface of the FTO glass was treated with O_2_ plasma to modify its surface energy.

### 2.2. Fabrication of Photovoltaic Devices

The nickel oxide layer was utilized as the hole-selective layer in the OPV with a conventional structure. The BHJ active layer with a thickness of approximately 90 nm was spin-coated on the HTL. 2.5 wt % BHJ solution was prepared by mixing the donor and acceptor materials in a weight ratio of 1:1.5 in a mixture solvent comprising 3 v% of 1,8-diiodooctane (DIO) in chlorobenzene. The donor and acceptor materials were poly((4,8-bis[(2-ethylhexyl)oxy]benzo[1,2-b:4,5-b′]dithiophene-2,6-diyl)(3-fluoro-2-[(2-ethylhexyl)carbonyl]thieno[3,4-b]thiophenediyl)) (PTB7, purchased from 1-Material) and the fullerene derivative [6,6]-phenyl-C71-butyric acid methyl ester (PC_70_BM, purchased from Nano-C), respectively. The BHJ layers were dried in a vacuum chamber (at pressure lower than 10^−2^ Torr) for 2 h before coating the titanium oxide electron transport layer (approximately 6 nm thick). Next, an aluminum cathode (100 nm thick) was deposited by using a thermal evaporator (at pressure lower than 10^−6^ Torr). In the case of using the PEDOT:PSS interlayer between the nickel oxide and BHJ layers, a mixture solution of PEDOT:PSS (CLEVIOSTM AI 4083) and 2-propanol in the volume ratio 3:1 was spin-coated on the nickel oxide layer. Then, the spin-coated PEDOT:PSS layer was dried at 115 °C for 30 min on a hot plate.

### 2.3. Characterization

To investigate the surface morphology of the films, a field-emission scanning electron microscope (SEM) (JSM-7001F, JEOL Ltd.) and atomic force microscope (AFM) (XE-100, Park Systems.) were utilized in tapping mode. Device performance characterization was conducted to obtain the *J-V* curves by using a Keithley model 2400 source meter under simulated AM 1.5G 1 sun illumination, which was generated by a solar simulator Oriel Sol 3A (class AAA). The performance characterization was performed in a dry-room, where the controlled relative humidity was approximately 25%. A Si reference cell (Oriel P/N 91150V, VLSI standards) was used for adjusting the illuminated light (100 mW/cm^2^). The cell area was determined by using an aperture of area 11.43 mm^2^. The aperture was placed on top of the cell (of approximately 15 mm^2^). These area values were carefully characterized with a video microscope (Sometech, SV-35). The incident photon-to-current conversion efficiency (IPCE) was measured to obtain the external quantum efficiency (EQE). For this, a solar cell QE/IPCE measurement system (Zolix Solar Cell Scan 100) was utilized. The photoluminescence (PL) decay profile was characterized via the time-correlated single photon counting (TCSPC) measurement by using the second harmonic generation (SHG = 375 nm) of a tunable Ti:Sapphire laser with ~150 fs pulse width and the time-correlated single photon counting (TCSPC) module (PicoHarp, PicoQuant) at 20 K. A monochromator (SP-2150i, Acton.) was utilized with some collection optics to spectrally resolve the PL emission. 

## 3. Results and Discussion

**Figure 1:**
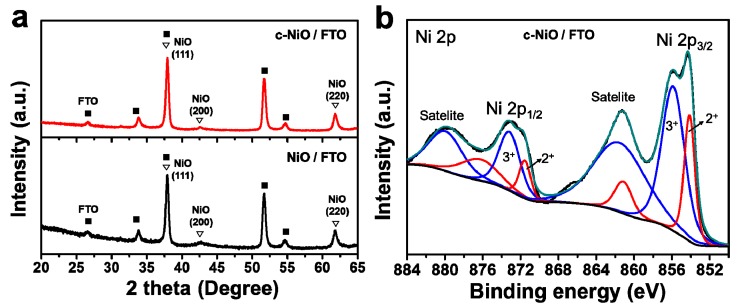
(**a**) X-ray powder diffraction (XRD) patterns (JCPDS# 04-0835) of the nickel oxide (NiO) layer coated fluorine-doped tin oxide (FTO) glass without using polyethylene glycol (PEG) (NiO/FTO) and compact nickel oxide layer coated FTO glass with PEG (c-NiO/FTO) and (**b**) Ni 2p XPS spectrum of c-NiO/FTO.

The fabricated NiO layers were composed of the nanocrystalline NiO structure, as shown in Figure 1a. The dominant (111), (200), and (220) peaks were well-assigned to the crystalline NiO [25,28]. No significant changes in the crystallinity were observed between the pristine NiO layer prepared by the PEG-free sol-gel process (NiO/FTO) and the compact NiO layer prepared by the PEG-assisted sol-gel process (c-NiO/FTO). For the sake of understanding the surface oxidation state of c-NiO/FTO, the Ni 2p XPS spectrum was characterized (Figure 1b), where two spin-orbit doublets and the two associated shakeup satellites were observed. The presence of multiple peaks assigned to Ni^2+^ (centered at 854.1 and 872.0 eV) and Ni^3+^ (centered at 856.1 and 873.9 eV) implies that the c-NiO/FTO was composed of the preferred nonstoichiometric NiO with a combination of two oxidation states (2+ and 3+) [29,30,31]. This also indicates that the quasi-localized holes on the Ni^2+^ vacancies in the NiO lattice create Ni^3+^ ions, enhancing the hole conductance in the HTL [24].

**Figure 2:**
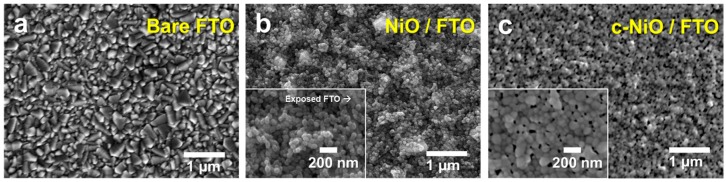
Scanning electron microscope (SEM) images of (**a**) bare FTO glass substrate, (**b**) nickel oxide layer coated FTO glass without using PEG (NiO/FTO), and (**c**) compact nickel oxide layer coated FTO glass with PEG (c-NiO/FTO).

The surface morphology of the NiO layer coated on the FTO glass was investigated, as shown in Figure 2. The pristine NiO layer prepared by the same sol-gel process without PEG shows a randomly assembled nanoparticulate film (Figure 2b). The *α*-terpineol causes the PEG-free sol-gel process to produce a mesoporous structure with globular nanoparticles (with a diameter of approximately 50 nm) [22]. However, the rough surface morphology was observed in NiO/FTO, which is ascribed to the high roughness of FTO. Further, the exposed FTO surface was observed because of the poor coverage of the pristine NiO layer. The PEG-assisted sol-gel process resulted in the compact NiO layer, which induced the modified surface morphology displayed in Figure 2c. Due to the stabilizing effect of PEG on the colloidal solution [32], the NiO nanoparticles were close-packed in a compact NiO layer, inducing the smooth morphology.

**Figure 3:**
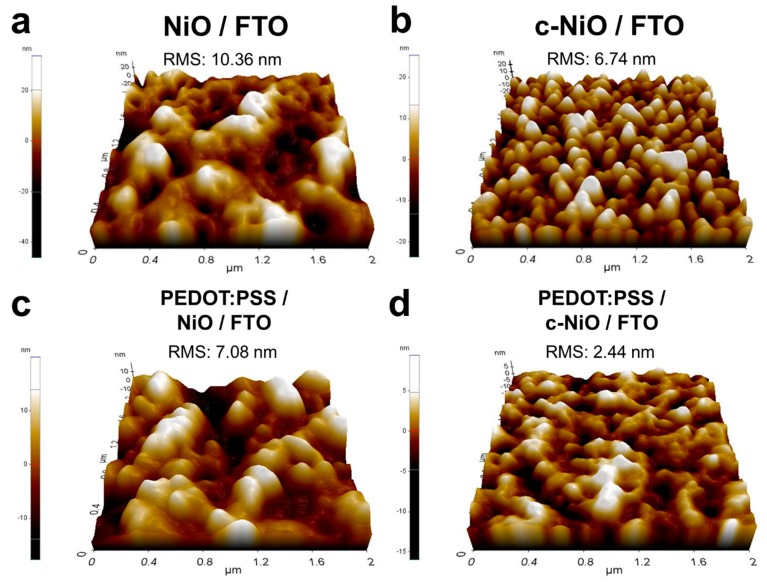
3D atomic force microscope (AFM) images of (**a**) nickel oxide on FTO (without PEG), (**b**) compact nickel oxide (c-NiO) on FTO (with PEG), (**c**) PEDOT:PSS coated nickel oxide on FTO, and (**d**) PEDTO:PTSS coated compact nickel oxide (c-NiO) on FTO.

Further investigation on the surface morphology was conducted with atomic force microscope (AFM) characterization (Figure 3). The high-resolution 3D AFM images (scale: 2 µm × 2 µm) demonstrate that the PEG-assisted sol-gel synthesis resulted in a much smoother surface morphology than that obtained in the PEG-free process. The RMS value was drastically reduced from 10.36 nm for NiO/FTO (Figure 3a) to 6.74 nm for c-NiO/FTO Figure 3b). It is noted that the metal oxide layers prepared by the solution-based process can have pinholes, which are formed by organic stabilizers or surfactants in the precursors. Tiny pinholes measuring less than few tens of nanometers in diameter are observed in c-NiO/FTO (Figure 2c), which are formed by the structure-determining agent, i.e., PEG. These tiny pinholes were successfully passivated and the uniform surface coverage was achieved by introducing PEDOT:PSS on the surface of NiO via simple spin-coating. As shown in Figure 3c, the rough surface of the pristine NiO layer hindered the formation of the thin PEDOT:PSS film. Therefore, the overlaid PEDOT:PSS had a rough surface morphology; its RMS value was 7.08 nm. However, the PEDOT:PSS coated c-NiO/FTO in Figure 3d shows an outstandingly smooth surface morphology. The significant reduction of RMS value from 6.74 nm for c-NiO/FTO to 2.44 nm for PEDOT:PSS/c-NiO/FTO implies that the conformal coating of PEDOT:PSS induced the pinhole passivation effectively. This low RMS value can be compared with that of the ITO-based interlayer. According to a previous report, the RMS value of the spin-coated PEDOT:PSS on the ITO glass was in the 2–3 nm range [13]. Considering the reported single granule size of PEDOT:PSS (i.e., a diameter of 20–50 nm), the smooth and compact surface morphology of c-NiO/FTO formed the uniform PEDOT:PSS overlayer with a high coverage, resulting in a polymer/metal oxide hybrid interlayer with modified morphology. 

**Figure 4:**
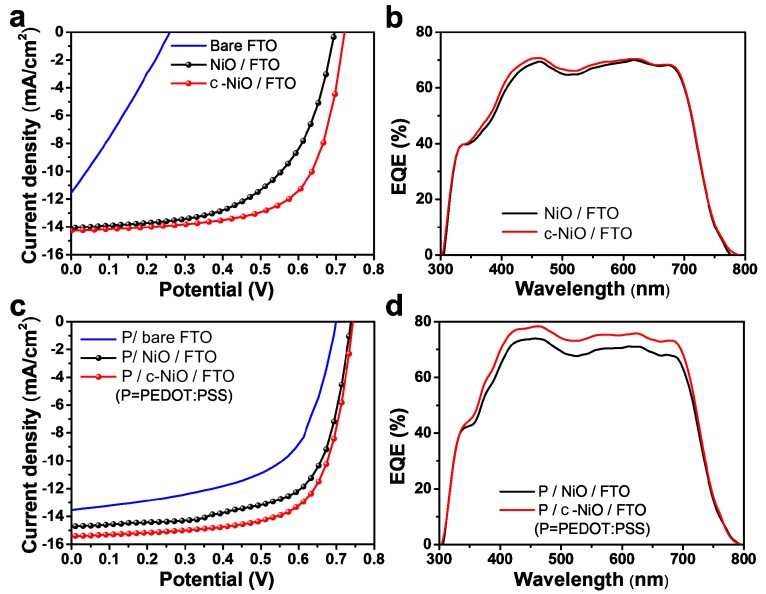
Current density-potential (*J-V*) curves (**a**) and external quantum efficiency (EQE) spectra (**b**) of OPVs comprising bare FTO, nickel oxide on FTO, and compact nickel oxide (c-NiO) on FTO, *J-V* curves (**c**) and EQE spectra (**d**) of OPVs comprising PEDOT:PSS on FTO, PEDOT:PSS coated nickel oxide on FTO, and PEDOT:PSS coated compact nickel oxide (c-NiO) on FTO.

The modified interfacial properties originating from the compact NiO layer resulted in the high-efficiency OPVs with conventional configuration of Al/TiO*_x_*/BHJ/HTL/FTO (Figure 4). The device performance parameters are summarized in Table S1 (Supplementary Information). The current density to potential (*J-V*) characteristics of the OPVs with bare FTO, NiO/FTO, and c-NiO/FTO are compared in Figure 4a. Interestingly, the significantly enhanced *V*_oc_ and *FF* parameters contributed to improving the PCE of the OPVs with c-NiO/FTO. The average PCE, *V*_oc_, and *FF* values were 6.91%, 0.722 V, and 66.98% for OPVs with c-NiO/FTO and 5.68%, 0.696 V, and 52.10% for OPVs with NiO/FTO, respectively. These enhancements in *V*_oc_ and *FF* can be attributed to the modified interfacial properties from the uniform and smooth morphology of the compact NiO interlayer, because these parameters were strongly affected by the contact resistances at the interface and the leakage currents in the metal oxide interlayers [33,34]. However, the tiny pinholes in c-NiO/FTO could not fully suppress the charge recombination, resulting in the slight improvement of the *J*_sc_ value from 14.06 mA/cm^2^ for NiO/FTO to 14.28 mA/cm^2^ for c-NiO/FTO. The IPCE values were also characterized, as shown in Figure 4b, where the slightly higher IPCE values of OPV with c-NiO/FTO were observed, well-matched with the slight increase in the *J*_sc_ parameter. Whereas all the performance parameters, including *V*_oc_, *J*_sc_, and *FF*, were remarkably enhanced in the OPVs with overlaid PEDOT:PSS on c-NiO/FTO (P/c-NiO/FTO), as shown in Figure 4c and Table S1 (Supplementary Information). Compared to the performance of the OPVs with PEDOT:PSS coated NiO/FTO (P/NiO/FTO), the *J*_sc_ parameter was outstandingly enhanced in the OPVs with P/c-NiO/FTO from 14.70 to 15.39 mA/cm^2^. This result indicates that the tiny pinholes were successfully passivated and the interfacial properties were ameliorated, suppressing the charge recombination. In addition, the modified interfacial properties of hole selective layer can influence the morphology of overlaid active layer as well. As shown in Figure S1 (Supplementary Information), the surface roughness of active layer was slightly reduced from 3.4 nm for the active layer coated on P/NiO/FTO to 2.6 nm for the active layer coated on P/c-NiO/FTO. This smooth surface morphology can promote the uniform formation of TiO_x_ interlayer and metal cathode on the active layer, which can contribute to the higher *J*_sc_ performance [35,36]. The improvement of the *J*_sc_ parameter was confirmed via IPCE characterization as shown in Figure 4d. The enhanced IPCE values of wavelength from 350 to 700 nm well-accounted for the high *J*_sc_ performance of the OPVs with P/c-NiO/FTO.

**Figure 5:**
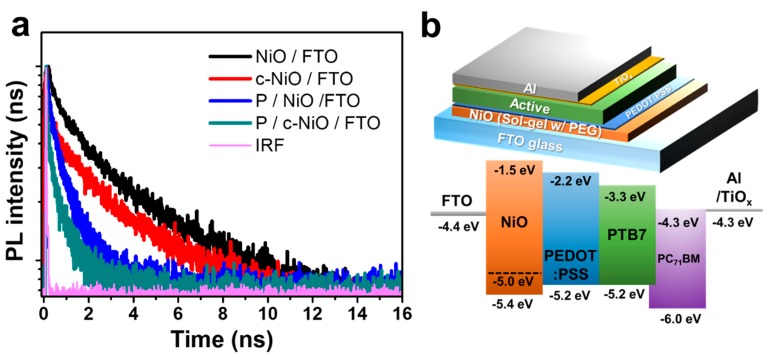
(**a**) Photoluminescence (PL) decay profiles from time-correlated single photon counting (TCSPC) measurement and (**b**) schematic illustration and energy diagram of OPV (IRF: instrument response function).

To understand the effects of the compact NiO interlayer on the charge extraction performance, the thermodynamic charge carrier migration behavior was investigated via TCSPC measurement. The characterized PL decay profiles are displayed in Figure 5a and fitted by a biexponential function. The time component parameters are summarized in Table S2 (Supplementary Information). The average PL decay lifetime (*τ*_avr_) was 1.659 ns for NiO/FTO and 0.582 ns for c-NiO/FTO. Approximately 2.8 times lower *τ*_avr_ indicates that the use of compact NiO can ameliorate the charge transportation property. Moreover, the significantly reduced *τ*_avr_, from 0.358 ns for P/NiO/FTO to 0.107 ns for P/c-NiO/FTO, confirms that the incorporation of the PEDOT:PSS and compact NiO interlayer facilitates efficient charge extraction. In addition, the remarkably reduced fraction of the slow component from 0.41 for P/NiO/FTO to 0.13 for P/c-NiO/FTO implies that the charge recombination originating from the traps at the spatially localized states was successfully suppressed [37,38,39,40]. Consequently, the hybrid HTL of PEDOT:PSS/compact NiO promoted efficient charge extraction with high selectivity of the hole migration as described in Figure 5b.

## 4. Conclusions

In summary, the use of facile PEG-assisted sol-gel synthesis of compact NiO film provided an effective strategy for introducing an effective hole-selective layer. The significantly uniform and smooth surface morphology of the compact NiO modified the interfacial properties, thereby inducing favorable charge extraction and suppressing the charge recombination in OPVs. Furthermore, the incorporation of the overlaid PEDOT:PSS onto the compact NiO layer facilitated efficient cascade charge migration and passivated the tiny pinholes. Finally, the compact NiO layer enhanced all the parameters determining the power conversion efficiency, including *V*_oc_, *J*_sc_, and *FF*. As a result, the introduction of the compact NiO hole-selective layer improved the efficiency from 5.68% to 6.91%. For the OPVs with the hybrid of PEDOT:PSS and NiO, the efficiency was improved from 7.26% for pristine NiO to 7.93% for compact NiO. These results demonstrate the promising strategy of using the facile sol-gel method with the environmental benign PEG as the structure-determining agent to obtain a metal-oxide interlayer with modified interfacial properties and to ameliorate charge migration with high selectivity for achieving high-efficiency solar cells.

## Supplementary Materials

The supplementary materials are available online at http://www.mdpi.com/2073-4360/11/1/120/s1.
