# Supplementary material for: PEG-assisted Sol-gel Synthesis of Compact Nickel Oxide Hole-Selective Layer with Modified Interfacial Properties for Organic Solar Cells
Source: Polymers (Basel). 2019 Jan 11;11(1):120. doi: 10.3390/polym11010120 (PMC6401794; doi:10.3390/polym11010120)
Supplement: Supplementary file 1 [file polymers-11-00120-s001.pdf]

Supplementary information

PEG-assisted Sol-gel Synthesis of Compact Nickel Oxide Hole-selective Layer with Modified Interfacial Properties for Organic Solar Cells

Jung Kyu Kim[1]*

[1]School of Chemical Engineering, Sungkyunkwan University (SKKU), Seobu-ro 2066, Jangan-gu, Suwon 16419, Republic of Korea

***Correspondence: legkim@skku.edu; Tel.: +82-31-299-7254**

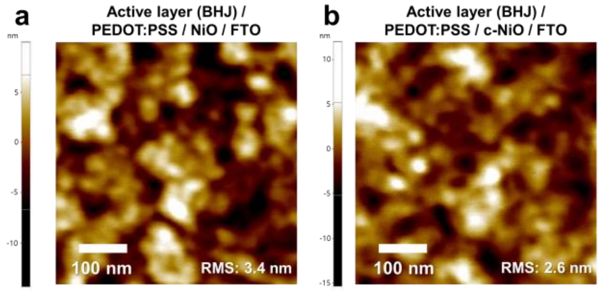

Figure S1. AFM images of active layers coated on PEDOT:PSS/nickel oxide/FTO (a) and PEDTO:PTSS/compact nickel oxide (c-NiO)/FTO (b).

Table S1. Photovoltaic parameters of OPVs.

**Devices**[1]	V_{oc} **(V)**	J_{sc} **(mA/cm^2)**	**FF (%)**	**PCE (%)**
Bare FTO	0.26	11.54	27.17	0.82
NiO/FTO	0.696 (±0.23)	14.06 (±1.58)	58.10 (±2.24)	5.68 (±1.32)
c-NiO/FTO	0.722 (±0.15)	14.28 (±0.61)	66.98 (±1.17)	6.91 (±0.83)
P (PEDOT:PSS)	0.682	13.63	59.34	5.52
P/NiO/FTO	0.738 (±0.11)	14.70 (±0.44)	66.92 (±0.37)	7.26 (±0.65)
P/c-NiO/FTO	0.744 (±0.08)	15.39 (±0.48)	69.23 (±0.55)	7.93 (±0.43)

[1] The average values were obtained from 10 devices for each condition except the OPVs prepared by bare FTO without any interlayer and PEDOT:PSS interlayer.

Table S2. Summary of PL decay lifetimes and fractional intensities of the samples. The average lifetime (τ_{avr}) was $\tau_1 f_1 + \tau_2 f_2$, where f1 and f2 are fractional intensities and τ_1 and τ_2 are lifetime.

**Devices composed of:**	**$\tau_1(f_1)$ [ns]**	**$\tau_2(f_2)$ [ns]**	**τ_{avr} [ns]**
NiO/FTO	0.514 (0.57)	3.169 (0.43)	1.659
c-NiO/FTO	0.082 (0.77)	2.255 (0.23)	0.582
P/NiO/FTO	0.137 (0.59)	0.675 (0.41)	0.358
P/c-NiO/FTO	0.046 (0.87)	0.519 (0.13)	0.107
